# Berufliche Infektion mit *Trichophyton verrucosum* bei einem Rinderzüchter

**DOI:** 10.1007/s00105-020-04655-7

**Published:** 2020-07-27

**Authors:** Uta Schumny, Cornelia Wiegand, Uta-Christina Hipler, Susanne Darr-Foit, Melanie Peckruhn, Silke Uhrlaß, Pietro Nenoff, Peter Elsner

**Affiliations:** 1 Klinik für Hautkrankheiten, Universitätsklinikum Jena, Erfurter Str. 35, 07743 Jena, Deutschland; 2 Labor für medizinische Mikrobiologie, Mölbiser Hauptstr. 8, 04571 Rötha, OT Mölbis, Deutschland

**Keywords:** Kälberflechte, Zoonose, Berufskrankheit, Pilzinfektion, Mykologische Untersuchung, Ringworm in cattle, Zoonosis, Occupational disease, Fungal skin diseases, Mycological typing techniques

## Abstract

Die Kälberflechte ist eine durch Tiere übertragene Pilzinfektion, die berufsbedingt auftreten und nach Nr. 3102 BKV (Berufskrankheiten-Verordnung) als Berufskrankheit anerkannt werden kann. Die durch *Trichophyton verrucosum* ausgelöste Zoonose zeichnet sich häufig durch einen schweren klinischen Verlauf aus, der nicht selten als bakterielle Infektion fehlgedeutet und primär antibiotisch behandelt wird. Die Gewinnung und mykologische Untersuchung von Schuppenmaterial ist diagnostisch entscheidend; auch eine Biopsie kann wegweisend sein. Die orale Therapie erfolgt leitliniengemäß mit Terbinafin. Zudem ist zum Schutz vor Reinfektionen auf besondere Hygienemaßnahmen in Ställen zu achten.

## Abstract (en)

Ringworm in cattle may cause an occupational skin disease in humans. *Trichophyton verrucosum* lead to a highly inflammatory fungal skin infection that is often misdiagnosed as bacterial disease and consequently mistreated with antibiotics. To establish the correct diagnosis, it is necessary to collect skin scales; in addition, a skin biopsy can be helpful. Deep dermatophyte infections by Trichophyton verrucosum can be treated effectively with oral terbinafine. In addition, it is necessary to pay careful attention to use suitable hygiene measures in the stables to protect against reinfection.

## Anamnese

Ein 31-jähriger Patient stellte sich mit seit 4 Monaten bestehenden juckenden Hautveränderungen vor. Ambulant war er von Ärzten unterschiedlicher Fachrichtungen mit Antimykotika, Antibiotika sowie Steroiden in unterschiedlicher Kombination und Anwendungsdauer behandelt worden. Eine extern erfolgte Hautbiopsie war für die Diagnosefindung nicht zielführend gewesen.

Jüngst zurückliegende Urlaube wurden in Österreich verbracht. Der Patient arbeitet auf seinem landwirtschaftlichen Hof mit Hühnern, Hunden, Hasen und Rindern.

Nach den verschiedenen ambulanten frustranen Therapieversuchen erfolgte die stationäre Aufnahme unter der Diagnose „therapieresistente und progrediente Follikulitiden unklarer Genese“.

## Befund

**Figure 1:**
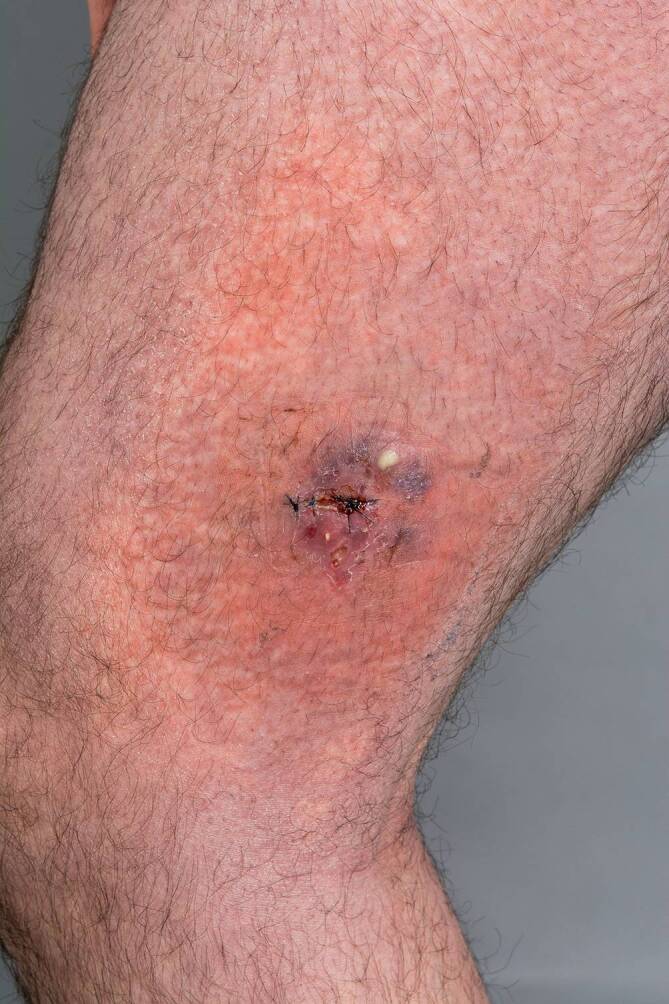

Am lateralseitigen rechten Oberschenkel zeigte sich eine etwa 50 mm messende, indurierte Plaque mit gruppiert stehenden lividen Nodi, teils mit erosiven Arealen. Umgebend fand sich eine diskrete weißliche Schuppung, randständig zudem eine 5 mm große, prall gelblich imponierende Pustel (Abb. 1). Weitere Läsionen in insgesamt milderer Ausprägung imponierten als unscharf und unregelmäßig begrenzte, tiefrote bis livide Plaques auf der gesamten unteren Extremität.

## Diagnose

**Figure 2:**
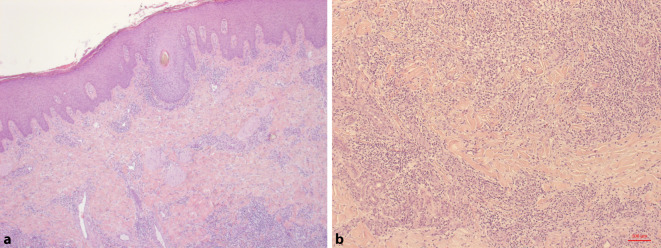

**Figure 3:**
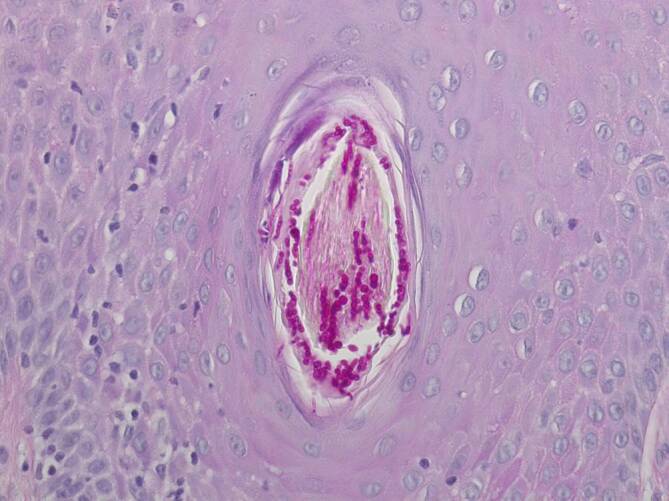

**Figure 4:**
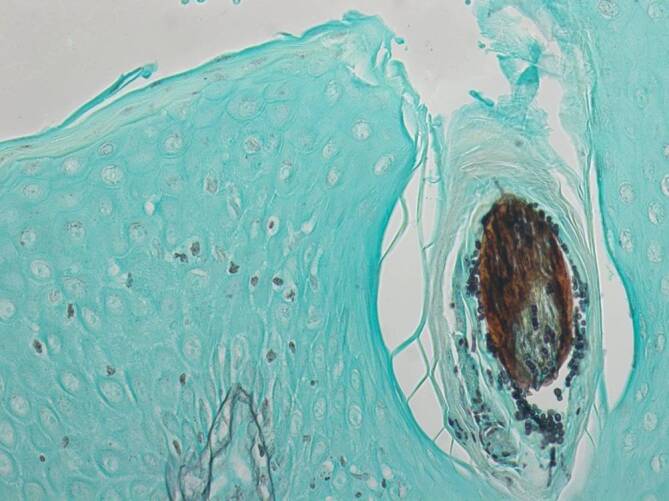

Aufgrund der hoch entzündlichen Aktivität der einzelnen Effloreszenzen wurden zunächst infektiöse Ursachen, insbesondere eine Pyodermie und eine kutane Tuberkulose sowie eine HIV(„human immunodeficiency virus“)-Infektion, ausgeschlossen. Des Weiteren konnte eine nichttuberkulöse Mykobakteriose molekularbiologisch ausgeschlossen werden. Ein Hautabstrich brachte den Nachweis normaler Standortflora. Die dermatohistologische Untersuchung einer tiefen Hautbiopsie ergab eine tiefe Dermatophytose (Tinea profunda) mit suppurativer, teils granulomatöser Entzündung (Abb. 2a, b). In den Spezialfärbungen, PAS(„periodic acid-Schiff“)-Reaktion (Abb. 3) und Grocott-Gomori-Silberfärbung (Abb. 4), zeigten sich massenhaft Hyphen im Follikelostium. Aufgrund der detaillierten Berufsanamnese war ein beruflicher Kontakt mit Rindern bekannt, sodass der Verdacht auf eine „Kälberflechte“ durch *Trichophyton verrucosum* nahelag.

Das durchgeführte Blankophor-Präparat („Fluorescent Brightener 28“, Sigma Aldrich, St. Louis, MO, USA) aus dem entnommenen Schuppenmaterial war negativ, und in der Pilzkultur zeigte sich lediglich eine Schimmelpilzbesiedlung. Die molekularbiologische Untersuchung des Materials mit einem Pan-Dermatophyten-Primerpaar erzielte einen positiven Dermatophytennachweis, allerdings ist die Speziesbestimmung mit dieser Methode nicht möglich. Eine artspezifische Dermatophyten-PCR (Polymerasekettenreaktion), bei der auf *Trichophyton* (*T.*) *rubrum, T. interdigitale, T. benhamiae* und Microsporum (*M.*) *canis* getestet wird, ergab jedoch wiederum ein negatives Ergebnis. Als zusätzliches Probenmaterial wurde Pilz-DNA (Desoxyribonukleinsäure) aus dem in Paraffin eingebetteten Hautbioptat isoliert. Die Sequenzierung der ITS(Internal Transcribed Spacer)-Region der r(ribosomale)DNA des Dermatophyten im Labor für medizinische Mikrobiologie Mölbis erbrachte hieraus schließlich den Nachweis von *T. verrucosum*. Aufgrund des beruflichen Rinderkontaktes wurde die Verdachtsanzeige einer BK (Berufskrankheit) 3102 (Von Tieren auf Menschen übertragbare Krankheiten) gestellt.

## Therapie und Verlauf

Trotz zunächst ausbleibenden Erregernachweises wurde aufgrund des klinischen Verdachts eine Therapie mit Terbinafin 250 mg 1‑mal täglich unter Kontrolle der Leberenzyme eingeleitet; zusätzlich wurde extern mit Ciclopiroxolamin-Creme behandelt. Vier Wochen später zeigten sich rückläufige livide bis bräunliche Plaques. Die Terbinafin-Behandlung wurde bis 3 Wochen nach Abheilung, insgesamt 14 Wochen, fortgeführt. Als Endbefund bestanden lediglich postinflammatorische Hyperpigmentierungen.

Zwei Jahre später stellte sich der Patient mit ähnlichem Hautbefund erneut vor. Nach seinem Bericht hatten weitere Mitarbeiter ähnliche Hautbefunde. Eine Impfung der Rinder sei 2 Jahre zuvor zwar erfolgt, bei regelmäßigem Zukauf sei dies jedoch nicht fortgeführt worden. Auch die Desinfektion der Gerätschaften sei in der Umsetzung kaum möglich gewesen. Zielgerichtet konnte ein Rezidiv diagnostiziert werden. Ein rascher Erfolg zeigte sich erneut unter systemischer Terbinafin-Therapie.

## Diskussion

Bei *T. verrucosum* handelt es sich um einen zoophilen Pilz, der in der Veterinärmedizin als Verursacher der wohl häufigsten Dermatose des Rindes, der sog. Kälberflechte, bekannt ist. Rinder sind das Hauptreservoir des Pilzes, selten wird er auch durch Hunde, Schweine, Schafe, Katzen, Ziegen und Pferde übertragen [9]. *T. verrucosum* kann direkt vom Tier auf den Menschen übertragen werden; häufige Lokalisationen sind dann exponierte Körperareale wie Arme, Hals und Gesicht [5]. Eine Übertragung kann ebenso über sporentragende Gerätschaften (beispielsweise Stallumfassungen, Bürsten) [6] stattfinden, selten jedoch auch von Mensch zu Mensch [9]. Im Leipziger Raum weisen 24 % der Rinder in betroffenen Ställen eine „Kälberflechte“ auf [1].

Bei Infektion dringt der keratophile Pilz über das Stratum corneum in die Haut ein und breitet sich entlang des Haarfollikels in die Tiefe aus. Aus einer anfangs oberflächlichen Entzündung entwickelt sich so ein für zoophile Erreger typisches, hoch inflammatorisches Krankheitsbild mit furunkuloiden Knoten, das gelegentlich auch mit Allgemeinsymptomen und Lymphknotenschwellung einhergeht. Aufgrund der stark entzündlichen Komponente wird nicht selten eine bakterielle Infektion angenommen und zunächst antibiotisch behandelt [8]. Zielführend ist neben der eigentlichen Diagnostik auch die ausführliche Berufs- und Sozialanamnese einschließlich Urlaub, Freizeit und Tierkontakten [3].

Der Erregernachweis kann, wie dieser Fall zeigt, mühsam sein. Als Untersuchungsmaterial dienen randständiges Schuppen- und epiliertes Haarmaterial. Bei Verwendung von epiliertem Haar aus einem entzündlichen Herd hätte die Sensitivität im vorliegenden Fall ggf. verbessert werden können. Ein konventionelles Nativpräparat mittels KOH (Kaliumhydroxidlösung) weist nur eine Sensitivität von 40–68 % auf, die teilweise durch Verwendung von Blankophor und Fluoreszenzmikroskopie gesteigert werden kann [7]. Auch die Pilzkultur weist eine eingeschränkte Sensitivität auf. Zudem ist eine Inkubationszeit von mindestens 4 Wochen nötig, um den überdurchschnittlich langsam wachsenden *T. verrucosum* zu bebrüten [7]. Der molekularbiologische Pilznachweis mittels PCR birgt gegenüber der Kultur 2 Vorteile: Die Sensitivität ist höher und die Zeit bis zur endgültigen Diagnosestellung kürzer [7]. Auch die Hautbiopsie kann bei Verwendung spezieller Färbungen hilfreich sein. Eine rasche Diagnostik ist insbesondere bei schweren Infektionen wichtig, um durch schnelle Therapieeinleitung Komplikationen zu verhindern. In Berichten werden abszedierende Verläufe sowie vernarbende Alopezien beschrieben [2]. Neuerdings ist auch der direkte Nachweis von *T.-verrucosum*-DNA in Formalin-fixierten und Paraffin-eingebetteten Hautgewebeschnitten mittels Sequenzierung der ITS-Region der Pilz-DNA möglich [10]. Die Methode liefert schnell Ergebnisse und ist hoch spezifisch.

Die Therapie tiefer Dermatophyteninfektionen sollte systemisch erfolgen. Terbinafin 250 mg täglich per os kann empfohlen werden. Bei hepatischer Metabolisation ist eine Lebererkrankung vor Erstgabe auszuschließen.

Gleichermaßen ist die Eruierung der Infektionsquelle erforderlich, die, wenn möglich, mitbehandelt werden sollte. Infizierte Tiere können mittels Enilconazol behandelt werden (die Verwendung von Terbinafin hat für Lebensmittellieferanten keine Zulassung). Gleichzeitig müssen die Stall- und Gerätedesinfektion erfolgen beispielsweise mittels 1 %igem Chlorkalk. Die Impfung der Tiere mit einer Lebendvakzine ist eine weitere Möglichkeit und dient sowohl der Prophylaxe als auch der Krankheitsverkürzung. Es konnte gezeigt werden, dass die Prävalenz der Kälberflechte bei flächendeckender Immunisierung gesenkt werden kann [4]. Die Tinea corporis profunda ist bei beruflichem Kontakt zu Rindern, beispielsweise bei Landwirten, eine anzeigepflichtige Berufserkrankung nach Nr. 3102 der Berufskrankheitenverordnung („Von Tieren auf Menschen übertragbare Erkrankungen“).

## Fazit für die Praxis

- Die Berufsanamnese kann für die richtige Diagnosestellung zielführend sein.
- Insbesondere zoophile Pilze lösen hoch entzündliche Befunde aus, die als bakterielle Infektionen fehlgedeutet werden können.
- Als Probenmaterial sollte bei tiefer Trichophytie neben Schuppen auch Haar Verwendung finden.
- Auch eine Biopsie kann zur Diagnose führen.
- Terbinafin stellt eine wirksame Therapie gegen *Trichophyton verrucosum* dar, die über einige Wochen täglich oral verabreicht werden sollte.

## References

[1] Bartosch T, Heydel T, Uhrlass S (2018). MALDI-TOF MS analysis of bovine and zoonotic trichophyton verrucosum isolates reveals a distinct peak and cluster formation of a subgroup with trichophyton benhamiae. Med Mycol.

[2] Blomer RH, Keilani N, Faber A (2012). Tinea capitis profunda due to trichophyton verrucosum with cMRSA superinfection in an infant. Hautarzt.

[3] Kirsten H, Haiduk J, Nenoff P (2019). Tinea barbae profunda due to trichophyton mentagrophytes : case report and review. Hautarzt.

[4] Lund A, Bratberg AM, Naess B (2014). Control of bovine ringworm by vaccination in Norway. Vet Immunol Immunopathol.

[5] Maslen MM (2000). Human cases of cattle ringworm due to trichophyton verrucosum in Victoria, Australia. Australas J Dermatol.

[6] Nenoff P, Handrick W, Kruger C (2012). Dermatomycoses due to pets and farm animals : neglected infections?. Hautarzt.

[7] Nenoff P, Krüger C (2012). Dermatophyten-Infektionen der Haut, Haare und Nägel – ein Update. Aktuelle Derm.

[8] O’Gorman SM, Britton D, Collins P (2015). An uncommon dermatophyte infection: two cases of cutaneous infection with trichophyton verrucosum. Clin Exp Dermatol.

[9] Roman C, Massai L, Gianni C (2001). Case reports. Six cases of infection due to trichophyton verrucosum. Mycoses.

[10] Wollina U, Hansel G, Uhrlass S (2018). Deep facial mycosis due to trichophyton verrucosum-molecular genetic identification of the dermatophyte in paraffin-embedded tissue-case report and review of the literature. Mycoses.

